# Depression comorbid with tuberculosis and its impact on health status: cross-sectional analysis of community-based data from 48 low- and middle-income countries

**DOI:** 10.1186/s12916-017-0975-5

**Published:** 2017-11-28

**Authors:** Ai Koyanagi, Davy Vancampfort, André F. Carvalho, Jordan E. DeVylder, Josep Maria Haro, Damiano Pizzol, Nicola Veronese, Brendon Stubbs

**Affiliations:** 1 Research and Development Unit, Parc Sanitari Sant Joan de Déu, Universitat de Barcelona, Fundació Sant Joan de Déu, Dr. Antoni Pujadas, 42, Sant Boi de Llobregat, Barcelona, Spain; 2 Instituto de Salud Carlos III, Centro de Investigación Biomédica en Red de Salud Mental, CIBERSAM, Madrid, Spain; 3 KU Leuven Department of Rehabilitation Sciences, Leuven, Belgium; 4 KU Leuven, University Psychiatric Center KU Leuven, Leuven-Kortenberg, Belgium; 5 Department of Clinical Medicine and Translational Psychiatry Research Group, Faculty of Medicine, Federal University of Ceará, Fortaleza, Brazil; 6 Fordham University, Graduate School of Social Service, New York, NY USA; 7 Operation Research Unit, Doctors with Africa, Maputo, Mozambique; 8 Institute for Clinical Research and Education in Medicine (IREM), Padova, Italy; 9 National Research Council, Neuroscience Institute, Aging Branch, Padova, Italy; 10 Physiotherapy Department, South London and Maudsley NHS Foundation Trust, Denmark Hill, London, UK; 11 Health Service and Population Research Department, Institute of Psychiatry, Psychology and Neuroscience, King’s College London, London, UK; 12 Faculty of Health, Social Care and Education, Anglia Ruskin University, Chelmsford, UK

**Keywords:** Tuberculosis, Depression, Low- and middle-income countries, Epidemiology

## Abstract

**Background:**

Depression in tuberculosis increases the risk for adverse health outcomes. However, little is known about comorbid depression and tuberculosis in the general population. Thus, we assessed the association between depression and tuberculosis, and the decrements in health status associated with this comorbidity in 48 low- and middle-income countries.

**Methods:**

Cross-sectional, community-based data from the World Health Survey on 242,952 individuals aged ≥ 18 years were analyzed. Based on the World Mental Health Survey version of the Composite International Diagnostic Interview, past 12-month depression was categorized into depressive episode, brief depressive episode, subsyndromal depression, and no depression. Health status across six domains (cognition, interpersonal activities, sleep/energy, self-care, mobility, pain/discomfort) was assessed. Multivariable logistic and linear regression analyses were performed to assess the associations.

**Results:**

The prevalence of depressive episode among those with and without tuberculosis was 23.7% and 6.8%, respectively (*P* < 0.001). Tuberculosis was associated with a 1.98 (95% CI 1.47–2.67), 1.75 (95% CI 1.26–2.42), and 3.68 (95% CI 3.01–4.50) times higher odds for subsyndromal depression, brief depressive episode, and depressive episode, respectively. Depressive episode co-occurring with tuberculosis was associated with significantly worse health status across all six domains compared to tuberculosis alone. Interaction analysis showed that depression significantly amplifies the association between TB and difficulties in self-care but not in other health domains.

**Conclusions:**

Depression is highly prevalent in adults with tuberculosis, and is associated with worse health status compared to tuberculosis without depression. Public health efforts directed to the recognition and management of depression in people with tuberculosis may lead to better outcomes.

**Electronic supplementary material:**

The online version of this article (doi:10.1186/s12916-017-0975-5) contains supplementary material, which is available to authorized users.

## Background

Tuberculosis (TB) is one of the top 10 causes of deaths globally [1]. In 2015, there were 10.4 million new TB cases and 1.8 million deaths due to TB. Over 95% of TB cases and deaths occur in developing countries [1]. Depression often coexists with TB [2], and this comorbidity is associated with poor adherence to TB treatment and higher mortality [3]. Lack of adherence to anti-TB regimens may lead to higher risk for drug resistance, morbidity, and mortality, as well as community exposure to TB [4, 5].

In low- and middle-income countries (LMICs), the prevalence of depression is high and may be increasing [6]. A recent large prospective study from Korea found that depression at baseline is associated with a higher risk for incident TB [7]. Depression may lead to an increased susceptibility to TB by compromising immunity or through neglected self-care [8]. Thus, depression may be an unrecognized driver of the global TB and multidrug resistant TB (MDR-TB) epidemics [2]. However, the few previous studies on the association between depression and TB from LMICs have only been conducted in clinical settings with small sample sizes, and information from the general population is lacking. Furthermore, there is limited information on the joint effect of TB and depression on health status. We therefore assessed the association between TB and depression, and whether the co-occurrence of TB and depression confers a more pronounced decrement in health status and function compared to TB alone using community-based, predominantly nationally representative data from 48 LMICs that participated in the World Health Survey (WHS). Epidemiological data on the TB/depression comorbidity and its effect on health outcomes are crucial to provide a more accurate assessment of the public health significance of this comorbidity.

## Methods

### The survey

The WHS was a cross-sectional survey carried out in 70 countries from 2002 to 2004. Survey details are available elsewhere (http://www.who.int/healthinfo/survey/en/). Briefly, single-stage random sampling and stratified multi-stage random cluster sampling was conducted in 10 and 60 countries, respectively. Eligible participants were those with a valid home address and aged ≥ 18 years. One individual was randomly chosen from the household with the use of Kish tables. The questionnaire was subject to standard translation procedures to ensure comparability between countries. Face-to-face interviews were conducted by trained interviewers. The overall individual response rate was 98.5% [9]. To adjust for non-response, sampling weights were generated using the population distribution as reported by the United Nations Statistical Division. Ethical approval for the survey was provided by ethical boards at each study site. All participants gave their informed consent.

### Variables

#### TB

Because the WHS did not include mycobacterial culture or sputum smear examinations, TB was based on past 12-month symptoms of active TB. Specifically, as in previous WHS publications [10–12], those who had both (1) a cough that lasted for 3 weeks or longer and (2) blood in phlegm (or coughed up blood) were considered to have active TB. Previous studies have shown that the presence of these typical symptoms are likely to have a sensitivity and specificity of 65–70% and 55–75%, respectively, in the detection of TB [10].

#### Depression

The severity of depressive symptoms was established based on the individual questions of the World Mental Health Survey version of the Composite International Diagnostic Interview, which assessed the duration and persistence of depressive symptoms in the past 12 months [13]. Following the algorithms used in a previous WHS publication [14], four mutually exclusive groups were established based on the ICD-10 Diagnostic Criteria for Research (ICD-10-DCR) [15], where criterion B referred to symptoms of depressed mood, loss of interest, and fatigability. The algorithms used to define the four mutually exclusive groups were the following:

- *Depressive episode group*
  - At least two criterion B symptoms with a total of at least four depressive symptoms lasting 2 weeks most of the day or all of the day.
- *Brief depressive episode group*
  - Same criteria as depressive episode but did not meet the 2-week duration criterion.
- *Subsyndromal depression*
  - At least one criterion B symptom with the total number of symptoms being three or less. The criteria of duration of at least 2 weeks and presence of symptoms during most of the day had to be met.
- *No depressive disorder group*
  - None of the above.

In some analyses, we also dichotomized this variable as the absence or presence of depressive episode.

#### Health status

Health status was assessed with the use of 12 health-related questions pertaining to six different domains, namely (1) mobility, (2) pain and discomfort, (3) self-care, (4) cognition, (5) interpersonal activities, and (6) sleep and energy. These domains correspond to frequently used health outcome measures including the Short Form 12 [16], the Health Utilities Index Mark 3 [17], and the EuroQol 5D [18], and have been used as indicators of health status in prior WHS studies [19, 20]. Each domain consisted of two questions that assessed health function in the past 30 days. The actual questions can be found in Additional file 1: Table S1. Each item was scored on a five-point scale ranging from ‘none’ to ‘extreme/cannot do’. For each separate domain, we used factor analysis with polychoric correlations to obtain a factor score which was later converted to scores ranging from 0 to 100 with higher values representing worse health function [20].

#### Control variables

The selection of the control variables were based on past literature [10]. Sociodemographic variables included age, sex, education (no formal education, primary education, secondary or high school completed, or tertiary education completed), wealth, household size, and setting (rural or urban). Principal component analysis based on 15–20 assets was conducted to establish country-wise wealth quintiles. Current smoking was dichotomized as ‘Yes’ and ‘No’. Respondents were asked how many standard drinks of any alcoholic beverage they had on each day of the past 7 days. Females who reported consuming at least four drinks, and males who reported consuming at least five drinks, on 1 or 2 days in the past 7 days were considered infrequent heavy drinkers, and respondents who drank these amounts at least 3 days in the past 7 days were considered frequent heavy drinkers. All other respondents, apart from lifetime abstainers, were considered non-heavy drinkers [21, 22]. Body mass index (BMI; kg/m^2^) was based on self-reported weight and height, and was categorized as < 18.5 (underweight), 18.5–24.9 (normal weight), 25.0–29.9 (overweight), and ≥ 30 (obese). Diabetes was based on self-reported diagnosis.

### Statistical analysis

Publically available data of the WHS included 69 countries. The data were nationally representative for all countries with the exception of China, Comoros, the Republic of Congo, Ivory Coast, India, and Russia. We excluded 10 countries as they lacked sampling information. A further 10 high-income countries were deleted as the focus of the study was on LMICs. Finally, Turkey was deleted due to lack of information on education and diabetes. Thus, a total of 48 countries, of which 21 (n = 105,286) and 27 (n = 137,666) were low-income and middle-income countries, respectively, at the time of the survey (2003) according to the World Bank, were included in the final sample. According to the United Nations’ classification system (http://unstats.un.org/unsd/methods/m49/m49regin.htm), these corresponded to 20 countries in Africa (n = 82,424), 6 in the Americas (n = 62,732), 13 in Asia (n = 81,633), and 9 in Europe (n = 16,163). Information on the individual countries is provided in Additional file 1: Table S2.

Statistical analyses were performed with Stata 14.1 (Stata Corp LP, College station, Texas). Descriptive analyses included unweighted Ns, and weighted proportions and means.

First, in order to assess the association between TB (exposure) and depression (subtypes; outcome), we conducted multivariable multinomial logistic regression analyses using the overall sample. We also assessed the association between TB (exposure) and depressive episode (outcome) using multivariable binary logistic regression while stratifying by region (Africa, Americas, Asia, Europe) or country income level (low-income, middle-income). For these stratified analyses, we could not assess all depression subtypes as the outcome as the number of individuals with TB was small in some subsamples.

Next, we created a four-category variable based on the presence or absence of depressive episode and TB, namely (1) no depression and no TB (n = 183,455); (2) depression without TB (n = 11,440); (3) TB without depression (n = 2617); and (4) TB with depression (n = 687), to assess whether TB with depression is associated with a larger decrement in health status as compared with TB alone. We conducted multivariable linear regression with this this four-category variable as the exposure and the six health status variables as the outcomes (mobility, pain/discomfort, self-care, cognition, interpersonal activities, sleep/energy). We also conducted age-stratified analyses to assess whether the TB and depressive episode comorbidity have different effects on health status by age groups. Age was categorized as 18–44 (young adults; 67.8%), 45–64 (middle-aged adults; 23.6%), and ≥ 65 (older adults; 8.6%) years, broadly representing distinct life stages [23]. In order to assess whether there is effect modification by depressive episodes in the association between TB and health status, we also conducted interaction analysis by including an interaction term in the model using the overall sample (TB × depressive episode). We did not conduct interaction analysis by age groups due to the small sample size and possibility for lack of statistical power.

All regression analyses were adjusted for age, sex, education, wealth, household size, location, smoking, alcohol consumption, BMI, diabetes, and country. Adjustment for country was performed by including dummy variables in the models, as in previous WHS publications [11, 19]. All variables were included in the models as categorical variables with the exception of age, household size, and the six variables on health status (continuous variables). The sample weighting and the complex study design were taken into account in all analyses. Results from the logistic and linear regression are presented as odds ratios (ORs) and b-coefficients, respectively, with 95% confidence intervals (CIs). The level of statistical significance was set at *P* < 0.05.

Under 10% of the data were missing for the variables used in the analysis with the exception of TB (17.7%), BMI (30.3%), and diabetes (12.6%). For the regression analyses, we conducted multiple imputation of missing values using the *mi* commands in Stata using chained equations (20 imputations) [24]. This method uses information from all other variables except the one being imputed to impute missing values. The variables included in the imputation model were the outcome and all other covariates [12]. The results based on complete case analysis were similar.

## Results

**Fig. 1:**
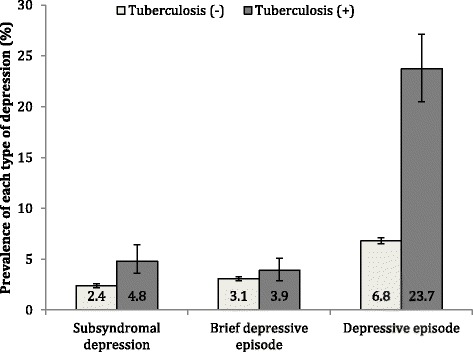
Prevalence of each type of depression by the presence or absence of tuberculosis. Bars denote 95% confidence intervals. Estimates are based on weighted sample

The analytical sample consisted of 242,952 individuals with a mean (SD) age of 38.4 (16.1) years and 50.8% were women (Table 1). The prevalence (95% CI) of TB was 1.7% (1.5–1.8%). All types of depression were more frequent among those with TB, with the difference being particularly pronounced for depressive episode (Fig. 1).

**Table 1:** Sample characteristics

Characteristic	Category	Unweighted N	% or Mean (SD)
Tuberculosis	No	196,417	98.3
	Yes	3347	1.7
Depression	No depression	205,752	87.7
	Subsyndromal depression	5238	2.6
	Brief depressive episode	6674	2.9
	Depressive episode	13,965	6.9
Age, years	Mean (SD)	233,879	38.4 (16.1)
Sex	Male	104,355	49.2
	Female	129,448	50.8
Education	No formal	52,116	26.5
	Primary	76,193	30.9
	Secondary	86,740	33.5
	Tertiary	17,860	9.2
Wealth	Poorest	51,599	20.1
	Poorer	45,893	20.0
	Middle	42,317	19.9
	Richer	40,128	20.0
	Richest	37,724	20.0
Household size	Mean (SD)	242,311	5.7 (3.0)
Setting	Rural	117,556	56.5
	Urban	114,825	43.5
Current smoking	No	174,814	73.5
	Yes	54,746	26.5
Alcohol consumption	Lifetime abstainer	142,282	66.4
	Non-heavy	74,016	28.8
	Infrequent heavy	8817	3.7
	Frequent heavy	2411	1.0
Body mass index, kg/m^2^	<18.5	16,883	13.8
	18.5–24.9	95,208	57.9
	25.0–29.9	38,700	19.3
	≥30.0	18,287	9.0
Diabetes	No	205,671	97.0
	Yes	6537	3.0

Data are unweighted N and weighted proportion or mean (SD)

*SD* standard deviation

The prevalence of depressive episode among those with and without TB was 23.7% (95% CI 20.5–27.1%) and 6.8% (95% CI 6.5–7.1%), respectively (χ^2^ test *P* < 0.001). The results of the multivariable multinomial logistic regression using the overall sample showed that TB is associated with a 1.98 (95% CI 1.47–2.67), 1.75 (95% CI 1.26–2.42), and 3.68 (95% CI 3.01–4.50) times higher odds for subsyndromal depression, brief depressive episode, and depressive episode, respectively (Table 2). Older age, female sex, lower levels of wealth, smoking, and diabetes were significant correlates of depressive episode.

**Table 2:** Association of tuberculosis and other covariates with depression estimated by multivariable multinomial logistic regression

		Depression subtypes (Reference = No depression)					
		Subsyndromal depression		Brief depressive episode		Depressive episode	
Characteristic	Category	OR (95% CI)	*P* value	OR (95% CI)	*P* value	OR (95% CI)	*P* value
Tuberculosis	Yes vs. No	1.98 (1.47–2.67)	<0.0001	1.75 (1.26–2.42)	0.0008	3.68 (3.01–4.50)	<0.0001
Age, years	Per unit increase	1.02 (1.02–1.03)	<0.0001	1.01 (1.00–1.01)	0.0003	1.02 (1.02–1.02)	<0.0001
Sex	Female vs. Male	1.84 (1.60–2.12)	<0.0001	2.19 (1.94–2.46)	<0.0001	2.06 (1.87–2.27)	<0.0001
Education	No formal	1.00		1.00		1.00	
	Primary	0.86 (0.72–1.03)	0.1101	0.95 (0.82–1.10)	0.4629	0.92 (0.83–1.03)	0.1414
	Secondary	0.76 (0.61–0.94)	0.0133	0.98 (0.81–1.18)	0.8148	0.77 (0.67–0.88)	0.0001
	Tertiary	0.83 (0.53–1.30)	0.4073	0.80 (0.62–1.02)	0.0686	0.82 (0.53–1.25)	0.3464
Wealth	Poorest	1.00		1.00		1.00	
	Poorer	1.00 (0.80–1.25)	0.9855	0.98 (0.84–1.16)	0.8384	0.95 (0.85–1.07)	0.4023
	Middle	1.07 (0.86–1.34)	0.5281	0.90 (0.76–1.06)	0.2022	0.96 (0.84–1.08)	0.4651
	Richer	1.05 (0.81–1.36)	0.7289	0.97 (0.82–1.16)	0.7557	0.87 (0.76–1.00)	0.0486
	Richest	0.90 (0.64–1.26)	0.5175	0.80 (0.66–0.97)	0.0229	0.69 (0.57–0.83)	0.0001
Household size	Per unit increase	1.03 (1.00–1.06)	0.0296	1.01 (0.98–1.03)	0.4946	1.01 (0.99–1.04)	0.2889
Setting	Urban vs. Rural	0.93 (0.79–1.11)	0.4373	1.12 (0.98–1.28)	0.0978	1.05 (0.94–1.17)	0.4040
Current smoking	Yes vs. No	1.31 (1.10–1.55)	0.0026	1.26 (1.10–1.44)	0.0007	1.29 (1.14–1.45)	<0.0001
Alcohol consumption	Lifetime abstainer	1.00		1.00		1.00	
	Non-heavy	1.27 (1.09–1.47)	0.0026	1.51 (1.32–1.73)	<0.0001	1.10 (0.99–1.22)	0.0685
	Infrequent heavy	1.45 (1.02–2.06)	0.0407	1.72 (1.29–2.28)	0.0002	1.03 (0.83–1.29)	0.7845
	Frequent heavy	2.10 (1.15–3.85)	0.0159	1.85 (1.18–2.92)	0.0080	1.14 (0.79–1.65)	0.4887
Body mass index, kg/m^2^	<18.5	1.02 (0.76–1.36)	0.8991	1.01 (0.83–1.23)	0.8988	1.08 (0.90–1.31)	0.4065
	18.5–24.9	1.00		1.00		1.00	
	25.0–29.9	1.10 (0.89–1.36)	0.3673	0.96 (0.83–1.11)	0.5485	0.99 (0.88–1.11)	0.8586
	≥30.0	1.09 (0.84–1.41)	0.5230	1.05 (0.86–1.29)	0.6136	1.05 (0.91–1.22)	0.4831
Diabetes	Yes vs. No	1.14 (0.84–1.54)	0.4000	1.39 (1.09–1.76)	0.0076	1.91 (1.62–2.24)	<0.0001

Model is adjusted for all variables in the Table and country

The association between TB and depressive episode estimated by multivariable binary logistic regression by regions or country income levels are shown in Table 3. TB was associated with a depressive episode across regions and county income levels although the estimates for Europe did not reach statistical significance, possibly due to lack of statistical power (OR, 2.67; 95% CI 0.75–9.52; *P* = 0.1293).

**Table 3:** Association between tuberculosis (exposure) and depressive episode (outcome) by regions or country income level

Region or country income level	OR (95% CI)	*P* value
Africa	3.50 (2.76–4.43)	<0.0001
Americas	2.74 (1.80–4.18)	<0.0001
Asia	3.75 (2.74–5.14)	<0.0001
Europe	2.67 (0.75–9.52)	0.1293
Low-income countries	3.52 (2.74–4.54)	<0.0001
Middle-income countries	3.24 (2.40–4.35)	<0.0001

Estimates are based on multivariable logistic regression

Models are adjusted for age, sex, education, wealth, household size, location, smoking, alcohol consumption, body mass index, diabetes, and country

*OR* odds ratio, *CI* confidence interval

Compared to those with no TB or depressive episode, depression alone, TB alone, and comorbid TB/depression were all significantly associated with worse health status scores in all domains. Comorbid TB/depression was associated with the largest decline (Table 4).

**Table 4:** Association between TB/depressive episode groups and health status estimated by multivariable linear regression

	TB (-) Depression (+)		TB (+) Depression (-)		TB (+) Depression (+)	
	b-coefficient (95% CI)	*P* value	b-coefficient (95% CI)	*P* value	b-coefficient (95% CI)	*P* value
Mobility	15.92 (14.81–17.04)	<0.0001	8.63 (6.55–10.71)	<0.0001	27.80 (23.60–31.99)	<0.0001
Self-care	11.96 (10.78–13.13)	<0.0001	5.34 (3.20–7.48)	<0.0001	23.80 (19.11–28.49)	<0.0001
Pain/discomfort	18.70 (17.39–20.00)	<0.0001	10.27 (8.24–12.30)	<0.0001	30.41 (26.97–33.84)	<0.0001
Cognition	16.55 (15.26–17.83)	<0.0001	8.63 (6.35–10.91)	<0.0001	24.24 (20.19–28.28)	<0.0001
Interpersonal activities	12.69 (11.53–13.86)	<0.0001	4.41 (2.34–6.48)	<0.0001	19.45 (14.81–24.09)	<0.0001
Sleep/energy	19.61 (18.37–20.85)	<0.0001	10.32 (7.94–12.70)	<0.0001	29.99 (25.30–34.68)	<0.0001

Reference category is TB (-) Depression (-)

Health status was the outcome and scores ranged from 0 to 100 with higher scores corresponding to worse health status

Models are adjusted for age, sex, education, wealth, household size, location, smoking, alcohol consumption, body mass index, diabetes, and country

*TB* tuberculosis, *CI* confidence interval

The results of the age-stratified analyses are shown in Additional file 1: Table S3. The decline in health status associated with depression alone and co-occurring TB/depression was similar across age groups, but that of TB alone was less pronounced in the oldest age group (i.e., ≥ 65 years). In order to assess whether the difference between TB alone and comorbid TB/depression is statistically significant, we also conducted the same analysis but changing the reference category to TB alone (overall sample). The b-coefficients (95% CIs) for comorbid TB/depression (vs. TB alone) were mobility 19.17 (14.52–23.81), self-care 18.46 (13.41–23.54), pain/discomfort 20.14 (16.23–24.04), cognition 15.61 (11.13-20.09), interpersonal activities 15.04 (10.13–19.96), and sleep/energy 19.67 (14.47–24.88) (all *P* < 0.0001). Overall, the interaction analysis showed that depression significantly amplifies the association between TB and difficulties in self-care but not with other health domains (Additional file 1: Table S4).

## Discussion

We found that TB is associated with the entire depression spectrum in the overall sample, and that the association between TB and depressive episode is comparable across regions and country income levels. Furthermore, the co-occurrence of depression and TB was associated with a major decrement in all health domains assessed compared to TB alone, with this additive effect being particularly pronounced for difficulties in self-care. The strengths of the study include the large sample size and use of predominantly nationally representative data from approximately one-fourth of the countries in the world obtained by standardized questionnaires across all countries. To the best of our knowledge, this is the first general population study on TB and depression. Furthermore, it is one of the very few studies assessing the association between TB and depression severity, and is the first to assess the joint effect of TB and depression on a variety of health conditions (i.e., mobility, self-care, pain/discomfort, cognition, interpersonal activities, sleep/energy). The finding that there may be a synergistic effect between TB and depression in terms of some health outcomes (i.e., self-care) is novel.

The association between TB and depression may be bidirectional [7, 25]. Depression itself may compromise immunity, leading to an increased risk for TB [8], while increased inflammation in TB may increase risk for depression [26]. Alternatively, depression may be a psychological reaction to the symptoms of TB (e.g., chronic cough, fatigue, weight loss) or associated disability [27], while hypoxia in chronic pulmonary diseases may induce depression [28]. It is also possible that patients with TB are perceived as a source of contagion in the community, which may lead to discrimination, stigma, social isolation, and rejection, and may predispose individuals to a higher risk for depression [27, 29]. Further, some anti-TB drugs can induce depression [27]. Finally, common risk factors, such as compromised immunity, stress, and malnutrition, may underlie the association [8, 26, 30, 31]. Regardless of whether depression and TB are etiologically related, the mere co-existence can complicate the diagnosis and management of these conditions, while it is also possible that they mutually influence each other and lead to the exacerbation of the other, altering the clinical course [27].

Other factors which were identified as significant correlates of a depressive episode in our study included sociodemographic factors (older age, female sex, lower levels of wealth), smoking, and diabetes. Previous studies have also found these factors to be associated with depression [32–36]. In particular, diabetes is known to increase risk for TB [37], and may be an important risk factor for TB in LMICs [10] as there is an upward trend in diabetes prevalence mainly driven by changes in lifestyles and diet in this setting [38]. On the other hand, diabetes and depression are often comorbid and common pathophysiological mechanisms (e.g., stress, inflammation) may underlie this co-occurrence [39].

In our study, compared to TB occurring in isolation, co-existing TB/depression was associated with decrements in all health domains assessed, while a significant interaction was observed for difficulties in self-care. These results are in line with a small cross-sectional study from Turkey showing that psychiatric comorbidity is associated with a higher rate of disability among TB patients [40]. Depression may lead to poor adherence to anti-TB drugs, and thereby exacerbate the symptoms of TB and its associated disability. Indeed, a prospective study from Peru showed that co-occurring TB/depression leads to lower adherence to TB treatment and higher mortality when compared to TB without depression [3]. The fact that a significant interaction was observed for self-care may imply that there is a synergistic effect between TB and depression. It may be hypothesized, for example, that depression leads to poor TB treatment adherence and exacerbation of symptoms, which in turn may lead to a worsening of depression. However, the precise underlying mechanisms or the reason why an interaction was only observed for self-care is unclear and warrants further investigation. Finally, delayed diagnosis of TB in people with depression may also partly explain our findings. It has been reported that delayed detection of physical diseases may be common in individuals with depression [41]. Thus, it may be that, when individuals with prior depression are diagnosed with TB, their TB symptoms are more severe compared to those without prior depression. Lack of motivation or social support and cognitive impairment, which may affect decision-making [42], might limit access to healthcare among depressed individuals, leading to delayed diagnosis and treatment initiation for TB.

Previous studies have shown that treating the psychological aspects of TB may lead to better clinical outcomes. For example, a prospective controlled trial in India showed that psychotherapy during TB treatment leads to higher adherence, treatment, and cure rates [43]. Furthermore, a psychological support group intervention for patients with MDR-TB in Peru showed that such an intervention can improve treatment adherence and completion [44]. Additionally, the formation of ‘TB clubs’ in Ethiopia increased treatment completion rates and reduced the stigma associated with TB [45]. Recently, a randomized controlled trial in Ethiopia showed that psychological counseling and educational intervention can substantially improve treatment adherence rates in TB [46].

A multi-faceted approach is likely to be relevant in addressing comorbid depression and TB in LMICs. First, previous studies from LMICs have shown that the treating doctor is often not aware of co-existing psychiatric morbidity in TB patients [47]. Thus, training of medical professionals and students on the psychological aspects of TB may lead to early detection and better management of psychiatric complications, and ultimately to a better clinical outcome of TB. Next, a close collaboration between TB and mental health specialists would be important for the early detection and treatment of depression in TB. Previous studies have shown that training of non-mental health specialists in LMICs may only have a limited impact on depression detection rates [48]. Thus, screening for depression may be a cost-effective strategy to improve detection rates of depression in TB. However, symptoms specific to depression (e.g., low mood, anhedonia) and symptoms of depression that overlap with TB (e.g., fatigue) should be distinguished. Some studies have assessed the validity of depression screeners such as the Center for Epidemiological Studies Depression scale or the General Health Questionnaire 12 among TB patients [40, 49]. These studies found that these screening tools can be used among TB patients to detect depression but that there may be a disease-specific optimal cut-off. Future studies on the validity and reliability of such screening tools are warranted, as only scarce data from limited populations are currently available. Finally, patient education and community awareness regarding facts and myths of TB may also be important [50], as discrimination and stigma can be underlying causes of depression in TB.

Our results should be interpreted in the light of several limitations. First, we lacked information on HIV, which is known to be associated with higher risk for TB [1] and depression [51]. Thus, some of the association may be attributable to comorbid HIV. However, this may not have been a major limitation as our region-wise analysis showed that TB is associated with depression even in areas with very low HIV prevalence (e.g., the Americas). Second, our study was based on the symptoms of TB rather than a laboratory confirmed diagnosis. Although we used the identical definition for TB used in previous publications [10–12], it is possible that some level of symptom overlap may exist between respiratory diseases such as pneumonia, bronchitis, and chronic obstructive pulmonary disease, which may also cause cough of long duration and hemoptysis. Thus, our estimates may partially be representing the association between these conditions and depression. Furthermore, the potential misclassification may have led to an underestimation of the association between TB and depression. However, it is reassuring that the prevalence of depression in TB was within the previously reported range of estimates among patients with confirmed TB [2]. Additionally, we are not aware of any other population-based data with such a large number of LMICs that can be used to investigate the TB–depression relationship. Third, high-risk groups, such as the institutionalized and homeless, were not included in our study and thus our findings are not generalizable to this population. Finally, the direction of causality cannot be established due to the cross-sectional design.

## Conclusions

In conclusion, individuals with TB have higher odds for depression, and the co-occurrence of TB and depression is associated with decrements in health. Screening for and addressing depression in individuals with TB may lead to better clinical outcomes. However, mental health services and specialists are limited in low-resourced settings where the highest burden of TB is located. Increased recognition of co-existing depression in TB patients by health professionals and the use of non-specialist health workers trained in mental healthcare, especially in resource-limited settings, may be key. However, given that healthcare workers are at increased risk of occupationally acquired TB in LMICs [52], sound infection control measures should be implemented to protect these individuals, yet this is a particular challenge in LMICs due to financial constraints. Finally, simultaneously addressing the mental and physical aspects of TB may lead to reduction in TB transmission [53], and also possibly in TB mortality and MDR-TB. This is an area for future research.

## Additional file

Additional file 1: Table S1. Questions used to assess health status. **Table S2.** Countries included in the analysis and sample size. **Table S3.** Association between TB/depressive episode groups and health status by age groups estimated by multivariable linear regression. **Table S4.** Interaction effect of TB and depressive episode on health status. (DOCX 45 kb)

## Abbreviations

BMI: body mass index
CI: confidence intervals
LMIC: low- and middle-income countries
MDR-TB: multidrug resistant tuberculosis
OR: odds ratio
TB: tuberculosis
WHS: World Health Survey
