# Supplementary material for: Depression comorbid with tuberculosis and its impact on health status: cross-sectional analysis of community-based data from 48 low- and middle-income countries
Source: BMC Med. 2017 Nov 28;15:209. doi: 10.1186/s12916-017-0975-5 (PMC5704363; doi:10.1186/s12916-017-0975-5)
Supplement: Additional file 1: Table S1. — Questions used to assess health status. Table S2. Countries included in the analysis and sample size. Table S3. Association between TB/depressive episode groups and health status by age groups estimated by multivariable linear regression. Table S4. Interaction effect of TB and depressive episode on health status. (DOCX 45 kb) [file 12916_2017_975_MOESM1_ESM.docx]

**Additional file 1**

| **eTable 1** Questions used to assess health status | |
| --- | --- |
| Mobility | (1) Overall in the last 30 days, how much difficulty did you have with moving around? |
|  | (2) In the last 30 days, how much difficulty did you have in vigorous activities, such as running 3 km (or equivalent) or cycling? |
| Self-care | (1) Overall in the last 30 days, how much difficulty did you have with self- care, such as washing or dressing yourself? |
|  | (2) In the last 30 days, how much difficulty did you have in taking care of and maintaining your general appearance (e.g. grooming, looking neat and tidy etc.) |
| Pain and discomfort | (1) Overall in the last 30 days, how much of bodily aches or pains did you have?  (2) In the last 30 days, how much bodily discomfort did you have? |
| Cognition | (1) Overall in the last 30 days, how much difficulty did you have with concentrating or remembering things? |
|  | (2) In the last 30 days, how much difficulty did you have in learning a new task (for example, learning how to get to a new place, learning a new game, learning a new recipe etc.)? |
| Interpersonal activities | (1) Overall in the last 30 days, how much difficulty did you have with personal relationship or participation in the community? |
|  | (2) In the last 30 days, how much difficulty did you have in dealing with conflicts and tensions with others? |
| Sleep and energy | (1) Overall in the last 30 days, how much of a problem did you have with sleeping, such as falling asleep, waking up frequently during the night or waking up too early in the morning? |
|  | (2) In the last 30 days, how much of a problem did you have due to not feeling rested and refreshed during the day (e.g. feeling tired, not having energy)? |

| **eTable 2** Countries included in the analysis and sample size | | | |
| --- | --- | --- | --- |
| Low-income countries | N | Middle-income countries | N |
| **Africa** |  | **Africa** |  |
| Burkina Faso | 4,948 | Mauritius | 3,968 |
| Chad | 4,870 | Morocco | 5,000 |
| Comoros | 1,836 | Namibia | 4,379 |
| Ethiopia | 5,089 | South Africa | 2,629 |
| Ghana | 4,165 | Swaziland | 3,117 |
| Ivory Coast | 3,251 | Tunisia | 5,202 |
| Kenya | 4,640 | **Americas** |  |
| Malawi | 5,551 | Brazil | 5,000 |
| Mali | 4,886 | Dominican Republic | 5,027 |
| Mauritania | 3,902 | Ecuador | 5,675 |
| Republic of Congo | 3,075 | Mexico | 38,746 |
| Senegal | 3,461 | Paraguay | 5,288 |
| Zambia | 4,165 | Uruguay | 2,996 |
| Zimbabwe | 4,290 | **Asia** |  |
| **Asia** |  | China | 3,994 |
| Bangladesh | 5,942 | Georgia | 2,950 |
| India | 10,687 | Kazakhstan | 4,499 |
| Laos | 4,988 | Malaysia | 6,145 |
| Myanmar | 6,045 | Philippines | 10,083 |
| Nepal | 8,820 | Sri Lanka | 6,805 |
| Pakistan | 6,501 | **Europe** |  |
| Vietnam | 4,174 | Bosnia Herzegovina | 1,031 |
|  |  | Croatia | 993 |
|  |  | Czech Republic | 949 |
|  |  | Estonia | 1,020 |
|  |  | Hungary | 1,419 |
|  |  | Latvia | 929 |
|  |  | Russia | 4,427 |
|  |  | Slovakia | 2,535 |
|  |  | Ukraine | 2,860 |

| **eTable 3** Association between TB/depressive episode groups and health status by age groups estimated by multivariable linear regression | | | | | | | | | | |
| --- | --- | --- | --- | --- | --- | --- | --- | --- | --- | --- |
|  |  | Age 18-44 years | | | Age 45-64 years | | | Age ≥65 years | | |
| Outcome | Exposure | b | 95%CI | P-value | b | 95%CI | P-value | b | 95%CI | P-value |
| Mobility | TB (-) Depression (+) | 15.18 | (13.48-16.89) | <0.0001 | 17.20 | (15.20-19.21) | <0.0001 | 15.40 | (13.24-17.56) | <0.0001 |
|  | TB (+) Depression (-) | 9.62 | (7.02-12.22) | <0.0001 | 10.39 | (6.77-14.00) | <0.0001 | 0.91 | (-6.04-7.85) | 0.7978 |
|  | TB (+) Depression (+) | 28.47 | (22.60-34.34) | <0.0001 | 29.59 | (21.88-37.31) | <0.0001 | 20.79 | (13.53-28.05) | <0.0001 |
| Self-care | TB (-) Depression (+) | 10.26 | (8.81-11.70) | <0.0001 | 12.68 | (10.76-14.60) | <0.0001 | 14.75 | (11.68-17.83) | <0.0001 |
|  | TB (+) Depression (-) | 5.69 | (3.35-8.03) | <0.0001 | 6.68 | (2.45-10.92) | 0.0020 | 0.03 | (-7.27-7.33) | 0.9936 |
|  | TB (+) Depression (+) | 21.88 | (15.80-27.95) | <0.0001 | 24.62 | (15.43-33.80) | <0.0001 | 25.24 | (17.44-33.05) | <0.0001 |
| Pain/discomfort | TB (-) Depression (+) | 18.87 | (17.14-20.59) | <0.0001 | 18.99 | (17.08-20.90) | <0.0001 | 16.61 | (14.20-19.02) | <0.0001 |
|  | TB (+) Depression (-) | 12.10 | (9.55-14.65) | <0.0001 | 10.21 | (6.70-13.72) | <0.0001 | 1.34 | (-4.52-7.20) | 0.6551 |
|  | TB (+) Depression (+) | 31.30 | (25.69-36.92) | <0.0001 | 30.95 | (25.65-36.26) | <0.0001 | 24.83 | (18.65-31.01) | <0.0001 |
| Cognition | TB (-) Depression (+) | 16.49 | (15.00-17.98) | <0.0001 | 16.54 | (14.05-19.03) | <0.0001 | 15.06 | (12.08-18.05) | <0.0001 |
|  | TB (+) Depression (-) | 9.40 | (6.72-12.08) | <0.0001 | 9.13 | (5.06-13.20) | <0.0001 | 3.56 | (-3.40-10.52) | 0.3160 |
|  | TB (+) Depression (+) | 26.41 | (20.45-32.37) | <0.0001 | 21.82 | (14.86-28.78) | <0.0001 | 21.50 | (15.69-27.32) | <0.0001 |
| Interpersonal activities | TB (-) Depression (+) | 11.81 | (10.22-13.40) | <0.0001 | 13.74 | (11.52-15.97) | <0.0001 | 12.71 | (9.61-15.82) | <0.0001 |
|  | TB (+) Depression (-) | 4.26 | (1.95-6.58) | 0.0003 | 5.34 | (0.93-9.75) | 0.0177 | 0.52 | (-6.59-7.64) | 0.8854 |
|  | TB (+) Depression (+) | 19.95 | (13.53-26.37) | <0.0001 | 15.71 | (7.78-23.64) | 0.0001 | 24.20 | (15.46-32.94) | <0.0001 |
| Sleep/energy | TB (-) Depression (+) | 19.21 | (17.53-20.88) | <0.0001 | 20.24 | (18.02-22.46) | <0.0001 | 18.29 | (16.03-20.55) | <0.0001 |
|  | TB (+) Depression (-) | 11.07 | (8.24-13.90) | <0.0001 | 11.36 | (7.13-15.58) | <0.0001 | 4.10 | (-1.84-10.05) | 0.1760 |
|  | TB (+) Depression (+) | 33.00 | (26.79-39.22) | <0.0001 | 27.99 | (19.43-36.54) | <0.0001 | 23.82 | (15.82-31.82) | <0.0001 |

Abbreviation: TB Tuberculosis; CI Confidence interval

Reference category is TB (-) Depression (-).

Health status was the outcome and scores ranged from 0 to 100 with higher scores corresponding to worse health status.

Models are adjusted for age, sex, education, wealth, household size, location, smoking, alcohol consumption, body mass index, diabetes, and country.

| **eTable 4** Interaction effect of TB and depressive episode on health status | | | | |
| --- | --- | --- | --- | --- |
| Outcome |  |  | b-coefficient (95%CI) | P-value |
| Mobility | Main effect | TB | 8.63 (6.55-10.71) | <0.0001 |
|  |  | Depression | 15.92 (14.81-17.04) | <0.0001 |
|  | Interaction | TB X Depression | 3.24 (-1.60-8.08) | 0.1897 |
| Self-care | Main effect | TB | 5.34 (3.20-7.48) | <0.0001 |
|  |  | Depression | 11.96 (10.78-13.13) | <0.0001 |
|  | Interaction | TB X Depression | 6.50 (1.22-11.79) | 0.0159 |
| Pain/discomfort | Main effect | TB | 10.27 (8.24-12.30) | <0.0001 |
|  |  | Depression | 18.70 (17.39-20.00) | <0.0001 |
|  | Interaction | TB X Depression | 1.44 (-2.67-5.54) | 0.4926 |
| Cognition | Main effect | TB | 8.63 (6.35-10.91) | <0.0001 |
|  |  | Depression | 16.55 (15.26-17.83) | <0.0001 |
|  | Interaction | TB X Depression | -0.94 (-5.61-3.73) | 0.6930 |
| Interpersonal | Main effect | TB | 4.41 (2.34-6.48) | <0.0001 |
| activities |  | Depression | 12.69 (11.53-13.86) | <0.0001 |
|  | Interaction | TB X Depression | 2.35 (-2.71-7.40) | 0.3625 |
| Sleep/energy | Main effect | TB | 10.32 (7.94-12.70) | <0.0001 |
|  |  | Depression | 19.61 (18.37-20.85) | <0.0001 |
|  | Interaction | TB X Depression | 0.06 (-5.13-5.26) | 0.9807 |

Abbreviation: TB Tuberculosis; CI Confidence interval

Health status was the outcome and scores ranged from 0 to 100 with higher scores corresponding to worse health status.

Models are adjusted for age, sex, education, wealth, household size, location, smoking, alcohol consumption, body mass index, diabetes, and country.
